# EgMIXTA1, a MYB-Type Transcription Factor, Promotes Cuticular Wax Formation in *Eustoma grandiflorum* Leaves

**DOI:** 10.3389/fpls.2020.524947

**Published:** 2020-10-22

**Authors:** Lishan Wang, Wanjie Xue, Xueqi Li, Jingyao Li, Jiayan Wu, Linan Xie, Saneyuki Kawabata, Yuhua Li, Yang Zhang

**Affiliations:** ^1^Key Laboratory of Saline-alkali Vegetation Ecology Restoration, Ministry of Education, College of Life Science, Northeast Forestry University, Harbin, China; ^2^College of Life Science, Northeast Forestry University, Harbin, China; ^3^Institute for Sustainable Agroecosystem Services, Graduate School of Agriculture and Life Science, The University of Tokyo, Tokyo, Japan

**Keywords:** cuticular wax, lipid biosynthesis, *Eustoma grandiflorum*, *MIXTA* genes, transgenic plants, water loss

## Abstract

In the aerial plant organs, cuticular wax forms a hydrophobic layer that can protect cells from dehydration, repel pathogen attacks, and prevent organ fusion during development. The *MIXTA* gene encodes an MYB-like transcription factor, which is associated with epicuticular wax biosynthesis to increase the wax load on the surface of leaves. In this study, the *AmMIXTA*-homologous gene *EgMIXTA1* was functionally characterized in the *Eustoma grandiflorum*. *EgMIXTA1* was ubiquitously, but highly, expressed in leaves and buds. We identified the *Eustoma* MIXTA homolog and developed the plants for overexpression. EgMIXTA1-overexpressing plants had more wax crystal deposition on the leaf surface compared to wild-type and considerably more overall cuticular wax. In the leaves of the overexpression line, the cuticular transpiration occurred more slowly than in those of non-transgenic plants. Analysis of gene expression indicated that several genes, such as *EgCER3*, *EgCER6*, *EgCER10*, *EgKCS1*, *EgKCR1*, and *EgCYP77A6*, which are known to be involved in wax biosynthesis, were induced by EgMIXTA1-overexpression lines. Expression of another gene, *WAX INDUCER1/SHINE1*, encoding a transcription factor that stimulates the production of cutin, was also significantly higher in the overexpressors than in wild-type. However, the expression of a lipid-related gene, *EgABCG12*, did not change relative to the wild-type. These results suggest that *EgMIXTA1* is involved in the biosynthesis of cuticular waxes.

## Introduction

*Eustoma grandiflorum* (*Raf.*) *Shinn.* is a perennial, herbaceous, ornamental plant, which originated from the southern part of North America (Wang et al., 2011). *E. grandiflorum*, one of the most common potted plants, has very high economic and ornamental value due to its large, attractive, long-stalked flowers, long-lasting in vase flowers (Ecker et al., 1994; Zaccai and Edri, 2002; Uddin et al., 2004; Lena et al., 2009). *E. grandiflorum*, used to produce cut flowers, is vulnerable to plant diseases, insect pest attacks, and require large amounts of water. Hence, considerable efforts were dedicated to screening for drought tolerance, disease, and insect pest resistance.

Cuticular waxes are composed of long chain fatty acids (VLCFAs) and their derivatives, including aldehydes, alkanes, esters, and primary and secondary alcohols (Kunst and Samuels, 2009; Bernard and Joubes, 2013). The cuticular waxes form the outermost barrier to non-stomatic loss of water and play a significant role in responding to environmental stress. Composition, secretion, and synthesis of wax and cutin are modulated during cell expansion (Suh et al., 2005) and vary in different tissues (Yonghua et al., 2007, 2009). Many genes involved in cuticular wax biosynthesis and transportation have been characterized by forward and reverse genetic approaches for understanding the metabolism of plant wax (Hooker et al., 2002; Aharoni et al., 2004; Kim et al., 2013). Some transcription factors (TFs) were also shown to regulate the biosynthesis of cuticle. For example, *Arabidopsis thaliana* WAX INDUCER1/SHINE1 (WIN1/SHN1), which belongs to the TF family of AP2/EREBP type, activates the genes of cuticular wax and cutin biosynthesis (Kannangara et al., 2007). *DECREASE WAX BIOSYNTHESIS* (*DEWAX*), another AP2 TF, represses the expression of cutic wax biosynthesis genes to negatively regulate wax production in Arabidopsis (Go et al., 2014).

The *MIXTA* gene, first identified in *Antirrhinum majus*, encodes a TF an R2R3 MYB, which is a key regulator for the differentiation of epidermal cells into conical cells (Noda et al., 1994). MIXTA and MIXTA-like TFs have been demonstrated to be critical regulators for epidermal cell differentiation across multiple species of plants (Brockington et al., 2013). The MIXTA/MIXTA-like TFs were documented to function as positive regulators for the formation of conical epidermal cells (Noda et al., 1994; Stilio et al., 2010), production of cotton fibers (Walford et al., 2011), trichomes (Gilding and Marks, 2010; Plett et al., 2010), and cuticle development (Oshima et al., 2013; Lashbrooke et al., 2015). The Arabidopsis MIXTA-like orthologs AtMYB16 and AtMYB106 have been shown to control the formation of cuticles, MYB106, and MYB16; loss- and gain-of-function research has shown that they control the expression of the genes of cutin biosynthesis (Oshima et al., 2013). AtMYB96 regulates cutin biosynthesis by directly binding to the promoters of genes involved in cuticular wax biosynthesis (Seo et al., 2011). *Solanum lycopersicum* MIXTA-like (SlMIXTA-like) positively regulates both cuticle and conical epidermal cell formation, and links cutin polymer formation, cuticle assembly, and epidermal cell patterning in the fruit (Lashbrooke et al., 2015). The *Artemisia annua* MIXTA-like protein AaMIXTA1 is a positive regulator of trichome initiation and cuticle development; it forms a regulatory complex leading to increased transcription of *AaHD1* and cuticle development genes (Yan et al., 2018). These studies indicate the involvement of MIXTA-like MYBs in cuticle development.

In this study, we functionally characterize the *E. grandiflorum MIXTA-like 1* (*EgMIXTA1*) gene by investigating the transgenic lines that overexpress *EgMIXTA1*. Our result indicated that *EgMIXTA1* participates in the regulation of cuticular wax biosynthesis genes in *E. grandiflorum*. Moreover, we suggest an essential role for an *EgMIXTA-like* gene and investigate the regulatory relationships between *EgMIXTA1* and other genes involved in the biosynthesis of cuticular wax.

## Materials and Methods

### Plant Materials and Growth Conditions

Seeds of *E. grandiflorum* cv. No. 2003-2-2, were used to generate the *MIXTA1* overexpression lines (OX-1, OX-2, and OX-3) The seeds were rinsed with sterilized water for 10 min, rinsed twice with 70% alcohol for 1 min each, surface sterilized with 3% (v/v) sodium hypochlorite for 10 min, and rinsed with sterilized water five times for 5 min each before sowing on 1.5% agar plates containing Murashige and Skoog (MS) medium. After sowing, the plates were kept at 4°C for 2 days and then incubated under aseptic conditions at 24°C with a photoperiod of 16 h white light (2,000 l×) In order to examine tissue-specific expression patterns, seeds were plated on MS medium with 1.5% sucrose and stratified in darkness for 2 days at 4°C before being transferred to the growth chamber (16 h of light and 8 h of darkness, 22°C). Roots, stems, leaves, petals, and buds were collected and stored at −80°C for further processing.

### Cloning of the Full-Length MIXTA1 Gene

Total RNA was extracted from leaves of the aseptic seedlings using TRIzol reagent (Invitrogen, Carlsbad, CA, United States), then treated with DNase I (TaKaRa, Otsu, Shiga, Japan) and re-extracted with TRIzol reagent. Reverse transcription PCR (RT-PCR) was performed using the SuperScript III Reverse Transcriptase (TaKaRa, Otsu, Shiga, Japan). PCR products were purified using the High Pure PCR Product Purification Kit (Invitrogen). A degenerate primer set was designed from protein domains of MIXTA conserved across proteins from related species, namely, *A. majus* (AY821655, AJ006292, X79108, AY661654), *Malus sieversii* (DQ074464), *Gossypium hirsutum* (AF336283), and *Petunia hybrida* (Z13996). Partial fragments of *EgMIXTA1* were amplified from the cDNA library by PCR. Then 5′ rapid amplification of cDNA ends (RACE) and 3′ RACE were performed with gene- and vector-specific primers [MIXTA1F/R, Primer5′ 1st (GSP1) Primer5′ 2nd (GSP2), Primer3′ 1st (GSP1), Primer3′ 2nd (GSP2), Adaptor, Anchor, Primer LF (5′ GSP), Primer LR (3′ GSP), MIXTA1-F/R] (Supplementary Table 1).

### Generation of Transgenic Plants

The coding sequence of *MIXTA1* from *E. grandiflorum* cv. No. 2003-2-2 cDNA was amplified using a standard RT-PCR protocol. The full-length coding region of *MIXTA1* was cloned into the binary vector pH7WG2D to generate an *MIXTA1* overexpression vector in which *MIXTA1* expression was driven by the CaMV35S promoter. The constructs were introduced into *Agrobacterium tumefaciens* strain LBA4404. Seedlings of *E. grandiflorum* (wild-type) were transformed using the leaf disc transformation method and screened on 0.8% agar plates containing diluted (50% v/v) MS medium and 20 mg/L kanamycin sulfate. Transgenic lines were selected based on kanamycin resistance.

### Real-Time Fluorescence Quantitative PCR Analysis

Total RNA was extracted from *E. grandiflorum* plants using TRIzol reagent (Invitrogen, Carlsbad, CA, United States). Two micrograms of total RNA pooled from three replicate extractions was used for reverse transcription with the PrimeScript First Strand cDNA Synthesis kit (TaKaRa, Tokyo, Japan). The cDNA was then diluted 20-fold, and expression was quantified using the Power SYBR Green PCR Master Mix with the 7500 Real-Time PCR System according to the manufacturer’s protocol (Applied Biosystems, Foster City, CA, United States). Amplification was done using the gene-specific primers listed in Supplementary Table 1. The quantitative RT-PCR (qRT-PCR) conditions used were 30 s at 95°C, followed by 40 cycles of 5 s at 95°C, 34 s at 60°C, and 15 s at 95°C. The relative quantification method (2^–ΔΔCT^) was used to evaluate quantitative variation between replicates. Data were normalized against the *EgActin* gene. The reactions were performed in biological triplicate using RNA samples extracted from three independent plant samples.

### Scanning Electron Microscopy

To observe the cuticular waxes in the transgenic aseptic seedlings, images were captured with a QUANTA 200 scanning electron microscopy (FEI Co., Hillsboro, OR, United States). When the seedlings were at least 5 weeks old, the third true leaves from each transgenic line were analyzed for cuticular wax. Before the observations, the leaves were cut into small pieces (1 cm × 1 cm) and fixed to sample holders, then dehydrated to make the surface of the leaves clean and water free. The samples were sputter-coated with gold particles using an SCD 005 Sputter Coater (Leica Co., Solms, Germany) and examined by scanning electron microscopy at an accelerating voltage of 10 kV.

### Immunostaining Analysis

Immunostaining was performed as previously described (Xuhong et al., 2007). Leaves were ground to a fine powder and fixed in ice-cold fixation buffer (4% formaldehyde, 10 mM Tris–HCl, pH 7.5, 10 mM EDTA, and 100 mM NaCl). Then, the sample was suspended in sorting buffer (100 mM Tris–HCl, pH 7.5, 50 mM KCl, 2 mM MgCl, 0.05% Tween 20, and 5% sucrose). Nuclei were sorted using a Falcon^®^ 40 μm Cell Strainer. Nuclei were incubated with antiEgMIXTA1 antibody, washed (10 mM sodium phosphate, pH 7.0, and 143 mM NaCl), incubated with Rhodamine Red-x-conjugated donkey anti-rabbit IgG (1/500 dilution; Alexa Fluor), and washed thoroughly. Images were captured and analyzed using Zeiss Axioskop 2 Plus, and processed using Adobe Photoshop software.

### Water Loss Assay

The rate of water loss was estimated using leaves from well-watered 5-week-old plants. The plants were acclimated in the dark for 6 h, then soaked in water for 1 h. Three of the third true leaves were then dried and weighed gravimetrically using a microbalance at the time points indicated.

## Results

### Identification and Sequence Analysis of the *EgMIXTA1* Gene

The experiments were performed to find the TFs that regulate the synthesis of cuticular waxes and the differentiation of epidermal cells in *E. grandiflorum*. We looked for the MIXTA-like gene encoding sequences in *A. majus* (AY821655, AJ006292, X79108, AY661654), *M. sieversii* (DQ074464), *G. hirsutum* (AF336283), and *P. hybrida* (Z13996) in GeneBank. Based on the conserved domains and RACE technology, we identified the *E. grandiflorum* homolog of the *MIXTA-like* gene and named it *EgMIXTA1*. *EgMIXTA1* has an ORF of 1,128 nucleotides, encoding a polypeptide of 375 amino acids.

**FIGURE 1:**
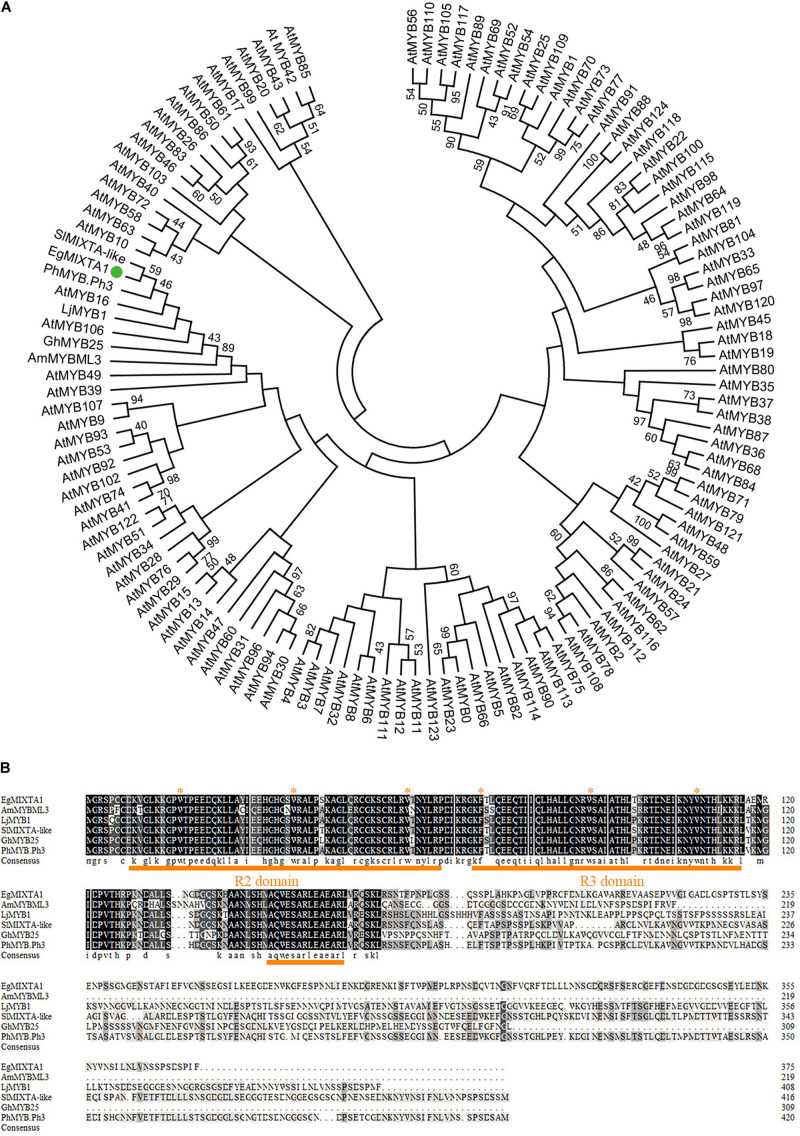
Sequence analysis of EgMIXTA1 homologs. **(A)** Phylogenetic analysis of EgMIXTA1, Arabidopsis R2R3 domain TFs, and other species of MIXTA-like proteins. ClustalW and MEGA7 were used to align the proteins and compute the neighbor-joining tree with significance percentages (bootstrap values = 1,000). **(B)** Amino acid alignment showing the conserved R2R3 MYB domain and subgroup 9 motifs underlined in orange. The highly conserved amino acid residues are marked with red stars.

Furthermore, the molecular phylogenetic analysis of EgMIXTA1 and Arabidopsis R2R3domain proteins showed that EgMIXTA1 is also closely related to AtMYB16 and AtMYB106 (Figure 1A). In previous phylogenetic analyses, it was found that AtMYB16 and AtMYB106 belong to the R2R3 subgroup 9 clade (SBG9); SBG9 members have a well-established relationship with trichome and papillate cell formation (Brockington et al., 2013). Compared with other MIXTA-like genes, EgMIXTA1 and *S. lycopersicum* MIXTA-like are more closely related; EgMIXTA1 shares 59% amino acid identity with *S. lycopersicum* MIXTA-like (Weng et al., 2011; Ying et al., 2019; Figure 1B). We were interested in whether EgMIXTA1 has same role in the differentiation of epidermal cells or some other function. Consequently, we examined the function of EgMIXTA1 in *E. grandiflorum* in greater detail.

### Expression Patterns of *EgMIXTA1* in *E. grandiflorum*

**FIGURE 2:**
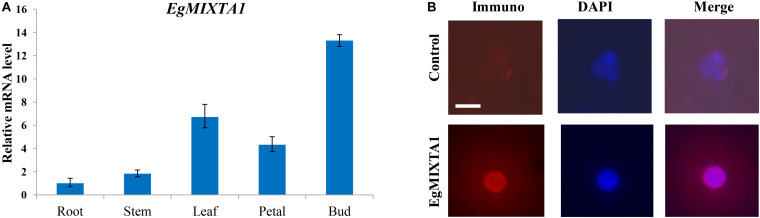
Gene expression pattern of *EgMIXTA1* and nuclear localization of EgMIXTA1 **(A)**. The expression levels of *EgMIXTA1* in roots, stems, leaves, buds, and petals of *E. grandiflorum* were measured by quantitative RT-PCR (qRT-PCR). Actin was used as an internal reference. Data are presented as mean ± SD (n = 3). **(B)** Nuclei isolated from the leaves of 5-week seedlings of *E Eustoma grandiflorum* were probed with anti-EgMIXTA1 or a solution lacking anti-EgMIXTA1 (control), and visualized by staining with DAPI (blue) and Rhodamine Red-x-conjugated donkey-anti-rabbit IgG. Scale bar, 5 μm.

In order to investigate the spatial expression pattern of *EgMIXTA1*, the levels of *EgMIXTA1* mRNA in different tissues were analyzed. In our study, qRT-PCR revealed that *EgMIXTA1* was ubiquitously expressed in roots, leaves, petals, stems, and flower buds (Figure 2A).

Nuclear localization is necessary for TFs to execute their functions, so we used nuclear immunostaining to determine whether EgMIXTA1 accumulates in the nuclei of *E. grandiflorum* leaf tissues. Nuclei isolated from *E. grandiflorum* leaves were stained with DAPI and anti-EgMIXTA1 or a control solution lacking antibodies, followed by incubation in Rhodamine Red-x-conjugated secondary antibody. The Rhodamine Red-x and DAPI signals overlapped in the nuclei stained with anti-EgMIXTA1, indicating that EgMIXTA1 is localized in the nucleus (Figure 2B).

### Overexpression of *EgMIXTA1* Increases Cuticular Wax Accumulation

**FIGURE 3:**
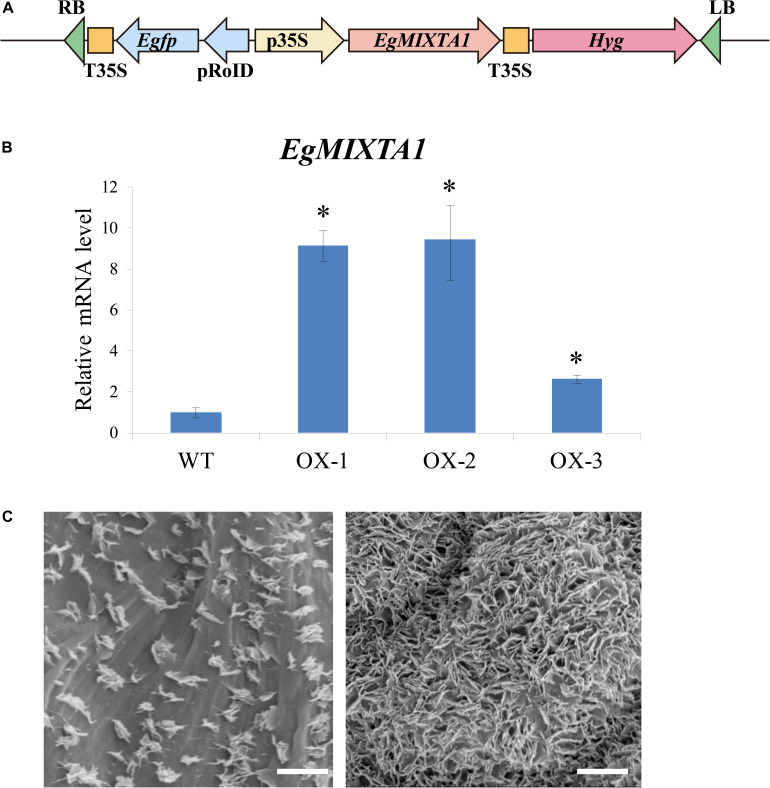
Cuticular wax accumulation in *E. grandiflorum* leaves with altered *EgMIXTA1* expression. **(A)** Schematic diagram of *EgMIXTA1* in the pH7WG2D vector under the control of the CaMV 35S promoter. p35S, CaMV 35S promoter; T-35S, CaMV 35S terminator; pRoID, rolD promoter; Egfp, enhanced green-fluorescent protein; Hyg, hygromycin-resistant gene; RB, T-DNA right border sequence; LB, T-DNA left border sequence. **(B)** Expression levels of *EgMIXTA1* in *EgMIXTA1*-overexpressing plants were measured by qRT-PCR. Total RNA was isolated from leaves of WT and MIXTA1 overexpression lines (OX-1, OX-2, and OX-3), and subjected to qRT-PCR analysis. Actin was used as an internal reference. Student’s *t*-test: ^∗^*P* < 0.01. **(C)** Scanning electron microscopy images of epicuticular wax crystals on the leaves of 3-week-old wild-type (left) and *MIXTA1* overexpression lines (right). Scale bar, 5 μm.

To further dissect the biological function of EgMIXTA1, transgenic *E. grandiflorum* plants overexpressing *EgMIXTA1* under the control of the 35S promoter were generated (Figure 3A). Three independent transgenic plants were obtained, and significantly increased levels of *EgMIXTA1* gene expression were detected in independent transgenic lines by qRT-PCR (Figure 3B). Scanning electron microscopy analysis showed obvious deposition of epicuticular wax crystals on the leaves of EgMIXTA1 overexpressing lines than wild-type (Figure 3C).

These results indicated that high amounts of cuticular waxes accumulated in the leaves of transgenic *E. grandiflorum* plants overexpressing *MIXTA1*.

### Expression of Wax Synthesis-Related Genes Is Altered in Leaves of Transgenic *E. grandiflorum* Plants Overexpressing *EgMIXTA1*

**FIGURE 4:**
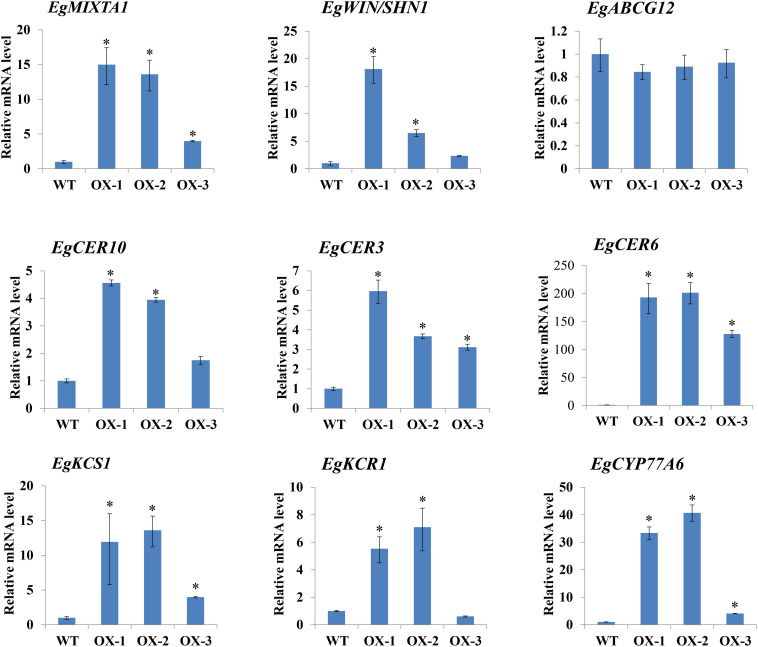
Expression of cuticular wax biosynthetic genes in *EgMIXTA1*-overexpressing plants. qRT-PCR analysis of the expression of genes involved in cutin and wax biosynthesis in the leaves of wild-type (WT) and *EgMIXTA1*-overexpressing plants (OX-1, OX-2, and OX-3). Expression level in the wild-type is set as 1. Actin was used as an internal reference. Student’s *t*-test: ^∗^*P* < 0.01.

Because epicuticular wax deposition was significantly increased in *EgMIXTA1*-overexpressing plants (Figure 3C), we asked whether the expression of wax biosynthetic genes was altered in these plants. The biosynthesis of cuticular wax is a complex process, and a number of enzymes related to cuticular wax biosynthesis and transport are involved in wax biosynthesis. C16 and C18 fatty acids are synthesized in the plastids then exported to the cytoplasm, where they are further elongated to VLCFAs through sequential addition of two-carbon units in a reaction catalyzed by fatty acid elongase complexes at the endoplasmic reticulum (Lee and Suh, 2013; Fich et al., 2016; Xue et al., 2017). We investigated the expression patterns of some important genes functioning in wax biosynthetic pathways. The *E. grandiflorum* genes were obtained by performing homologous sequence alignments with *Arabidopsis* genes. We selected six genes that might be involved in cuticular wax biosynthesis: *EgCER3*, *EgCER6*, *EgCER10*, *EgKCS1*, *EgKCR1*, and *EgCYP77A6* (Figure 4). We also checked the expression of a gene (*EgABCG12*) putatively involved in lipid transport. A previous study confirmed that the homolog of this gene in Arabidopsis, *ABCG12*, which encodes an ATP-binding cassette (ABC) transporter, is a key component of the export pathway for cuticular lipids (Bird et al., 2007; McFarlane et al., 2010). In addition, we investigated the expression pattern of *WIN1/SHN1*, a TF that induces cutin production in Arabidopsis (Kannangara et al., 2007). Real-time RT-PCR results showed that *EgCER3*, *EgCER6*, *EgCER10*, *EgKCS1*, *EgKCR1*, *EgCYP77A6*, and *EgWIN1* were upregulated in *EgMIXTA1*-overexpressing plants compared with non-transgenic plants. However, no significant changes in the transcript levels of *EgABCG12* were observed. This result revealed that overexpression of *EgMIXTA1* activated the expression of *E. grandiflorum* wax biosynthetic genes, which consequently caused an increase in cuticular wax biosynthesis and accumulation in transgenic *E. grandiflorum* plants.

### *EgMIXTA1*-Overexpressing Plants Lose Water More Slowly Than the Wild-Type

**FIGURE 5:**
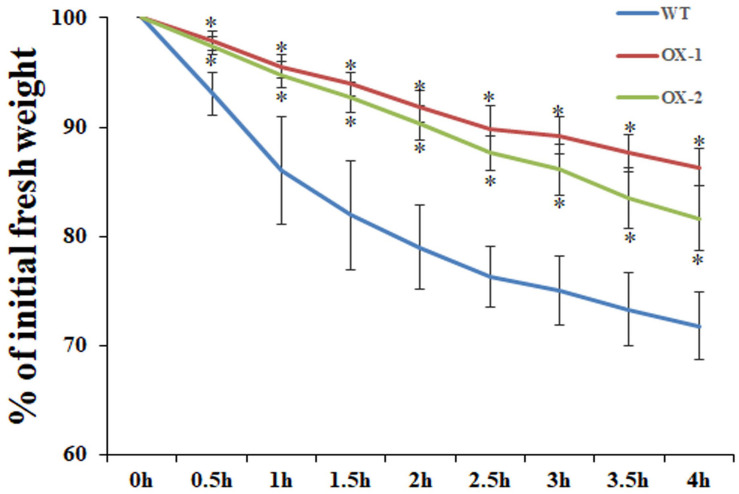
Water loss rate in the leaves of wild-type (WT), *35S:MIXTA1* (OX-1), and *35S:MIXTA1* (OX-2) plants. Leaves at the same developmental stage were excised and weighed at various time points after detachment. The water loss rate is shown as the percentage of initial fresh weight at each time point. The bars indicate the SE of three individual plants per genotype, and the asterisks denote significant differences from the wild-type (Student’s *t*-test, ^∗^*P* < 0.05).

Cuticular wax is the outermost layer that protects plants from water loss, and there is a lot of evidence showing that cuticular wax accumulation is closely associated with the water loss rate (Aharoni et al., 2004; Wang et al., 2018). We examined whether cuticular wax accumulation is linked to water loss by measuring cuticular transpiration. Five-week-old plants were dark acclimated for 6 h to ensure stomatal closure and soaked in water for 1 h in the dark to equilibrate the water content. Compared with wild-type leaves, transpiration occurred more slowly in the leaves of *EgMIXTA1*-overexpressing plants (Figure 5). The results showed that the elevated accumulation of cuticular waxes contributes to a reduction in water loss.

## Discussion

Plant cuticular wax covers the outer surface of aerial plant tissues. Many different studies have demonstrated that the plant cuticle plays an important role in limiting water loss and protecting plants from ultraviolet radiation and pathogen attacks (Kunst, 2003; Fich et al., 2016). Many *Arabidopsis* genes involved in wax synthesis, transport, and export, and in regulating the biosynthetic pathway have been identified. For example, *KCR1* encodes a β-ketoacyl-CoA reductase enzyme and is a candidate fatty acid elongase (Beaudoin et al., 2009), and *ECERIFERUM3* (*CER3*) is involved in the alkane-forming pathway (Chen et al., 2003). There are several lines of evidence from previous reports indicating that cuticular wax biosynthesis is regulated at the transcriptional level. For example, significant upregulation of the *WSD1*, *KCS2/DAISY*, *CER1*, *CER2*, *FAR3*, and *ECR* genes was observed in the leaves of transgenic Arabidopsis plants overexpressing MYB94 (Lee and Suh, 2015). In the activation-tagged myb96-1D mutant, a group of genes encoding cuticular wax biosynthetic enzymes, including *KCS1*, *KCS2*, *KCS6*, *KCR1*, *CER1*, and *CER3*, were greatly upregulated. In addition, the dominant active form of MYB106 (35S:MYB106-VP16) increased the expression of *WIN1/SHN1* and wax biosynthetic genes. In this study, we cloned the *EgMIXTA1* gene, which encodes a MIXTA-like R2R3-MYB TF, and generated transgenic plants overexpressing this gene. Overexpression of *EgMIXTA1* increased the expression of the wax biosynthetic genes (Figure 4).

MIXTA and MIXTA-like TFs have been shown to be key regulators of epidermal cell differentiation and cuticle biosynthesis. *A. majus*, a *mixta* mutant, in which conical cells become flat, shows alterations in petal color intensity (Noda et al., 1994). In Arabidopsis, the MIXTA-like genes AtMYB16 and AtMYB106 were shown to regulate epidermal cell morphology and cuticle development (Oshima et al., 2013). In this experiment, through scanning electron microscopy pictures, we observed that no epidermal hair was observed on the wild-type leaves of *E. grandiflorum*, and the same results were also shown after overexpression. At the same time, no changes were found in the morphology of the epidermal cells. In addition to the increase in cuticular wax, it is possible that EgMIXTA1 in the *E. grandiflorum* leaves mainly regulates the biosynthesis and transport of wax crystal. Unfortunately, we failed to observe changes in the morphological differentiation status and cuticular wax of *E. grandiflorum* petal epidermal cells after overexpression. In our study, EgMIXTA1 was demonstrated to function quite similarly to the MIXTA-like MYB TFs in determining cuticular wax formation in *E. grandiflorum* leaves (Figure 3C). It has been documented that biosynthesis and accumulation of cuticular waxes are closely linked with drought resistance responses. Under water deficit conditions, cuticular wax accumulation significantly increases. In roses, drought stress caused a 9–15% increase in wax load on leaves exposed to drought during development (Jenks et al., 2001). In tobacco, the total wax load increased 1.5- to 2.5-fold under periodic dehydration stress. In addition, a high-wax-producing *Dianthus spiculifolius* mutant with increased cuticular wax accumulation exhibited stronger drought resistance (Zhou et al., 2018). Our results demonstrate that EgMIXTA1 regulates cuticular wax deposition and decrease water loss (Figure 5).

*Eustoma grandiflorum*, which belongs to the Gentianaceae family, is becoming increasingly popular for use in cut flower production because it has large flowers, long stems, and an extended vase life (Davies et al., 1993). We found that elevated accumulation of cuticular waxes contributes to a reduction in water loss in *E. grandiflorum* (Figure 5). We did not conduct an experiment to elucidate the phenotype change and drought stress tolerance in the naturally growing conditions or the fresh-cut vase life. Our subsequent research will also verify this. The subsequent research will help in production and application. Based on this observation, increasing the accumulation of cuticular waxes may be important in reducing water loss and possibly delaying senescence and extending the vase life of cut *E. grandiflorum* flowers.

## Data Availability Statement

The datasets generated for this study are available on request to the corresponding author.

## Author Contributions

LW and JW carried out the experiments and data analysis and drafted the manuscript. YL and YZ provided guidance on experimental design and drafting the manuscript. WX, XL, JL, LX, and SK provided help in carrying out the experiments and modifying the manuscript. All authors contributed to the article and approved the submitted version.

## Conflict of Interest

The authors declare that the research was conducted in the absence of any commercial or financial relationships that could be construed as a potential conflict of interest.

## Supplementary Material

Supplementary Table 1 Primer sequences used in this paper.
