# Supplementary material for: EgMIXTA1, a MYB-Type Transcription Factor, Promotes Cuticular Wax Formation in Eustoma grandiflorum Leaves
Source: Front Plant Sci. 2020 Oct 22;11:524947. doi: 10.3389/fpls.2020.524947 (PMC7641950; doi:10.3389/fpls.2020.524947)
Supplement: Supplementary Table 1 — Primer sequences used in this paper. [file Table_1.DOCX]

Supplementary Material

**Table S1 Primer sequences used in this paper**

| Primer Name | Sequence (5′-3′) |
| --- | --- |
| *MIXTA*1F | 5′-AATTATTTGAGACCNGAYATHAA-3′ |
| *MIXTA*1R | 5′-TGGGTCACCGGRTCDATNCCCAT-3′ |
| Primer5’1^st^ (GSP1) | 5′-GTCACCGGATCTATTCCCATTTTG-3′ |
| Primer5’2^nd^ (GSP2) | 5′-GCAATTGCCGACCACCTGTTTCC-3′ |
| Primer3’1^st^ (GSP1) | 5′-GATGGCCAATCCAAACATGC-3′ |
| Primer3’2^nd^ (GSP2) | 5′-AAAGCGCCAGGCTCGAAG-3′ |
| Adaptor | 5′-GTACTAGTCGACGCGTGGCC-3′ |
| Anchor | 5′-GTACTAGTCGACGCGTGGCCTTTTTTTTTTTTTT-3′ |
| Primer LF (5′GSP) | 5′-ATGGGGCGTTCTCCATGTTG-3′ |
| Primer LR (3′GSP) | 5′-CTAAAATATTGGGGAATCAGAAGGT-3′ |
| q*MIXTA1*-F | 5′- GTTGGGTTCATCCCAGTCAA-3′ |
| q*MIXTA1*-R | 5′- CACTTGATGGGTTCTCGGAATAG-3′ |
| q *Actin*-F | 5′- CCGAGGCACCACTTAATCCAAAGGC -3′ |
| q *Actin*-R | 5′- TGCATGGGGCAGAGCATAACCCT -3′ |
| *EgMIXTA*o–F | 5′-ATGGGGCGTTCTCCATGTT-3′ |
| *EgMIXTA*o*–*R | 5′-CTAAAATATTGGGGAATCAGAAGGT-3′ |
| EgattB-*MIXTA*o-F | 5′-AAAAAGCAGGCTATGGGGCGTTCTCCATGTT-3′ |
| EgattB-*MIXTA*o-R | 5′-AGAAAGCTGGGTCTAAAATATTGGGGAATCAGAAGGT-3′ |
| attB-adaptor-F | 5′-GGGGACAAGTTTGTACAAAAAAGCAGGCT-3′ |
| attB-adaptor-R | 5′-GGGGACCACTTTGTACAAGAAAGCTGGGT-3′ |
| qWIN1/SHN1-F | TGCTCTGGTGGCGATTTACGCTG |
| qWIN1/SHN1-R | CGTTGCCTGACGCCTCTGAACTT |
| qCER10-F | ACAGACTGTTGCTGGTTATGT |
| qCER10-R | CTTCCGTCCTTCCCATCAAATA |
| qCER3-F | CTTGTGGTGTTTGCATCTTCTC |
| qCER3-R | CGATTCGACGAGTGCGATTA |
| qABCG12-F | TTCAGAGGGAAAGGATGAATGG |
| qABCG12-R | TAGCAGACAGTCCCAGAGATAA |
| qKCS1-F | CTGGTCCTCATGTCGTTGTTAG |
| qKCS1-R | TCTCTCTTCCTCTGGCTTGT |
| qKCR1-F | CATCCCGTCTGATCCTCTTTATG |
| qKCR1-R | CTGCACATTTATGCCGCTATTC |
| qCYP77A6-F | CCCACCGACGTATTTCTCTTT |
| qCYP77A6-R | CATTAGTCCCGACCGGAATATC |
| qCER6-F | GGCGAGGCGTTGAAATCTA |
| qCER6-R | TCCGGCCAATGAGTGTAAAG |

**
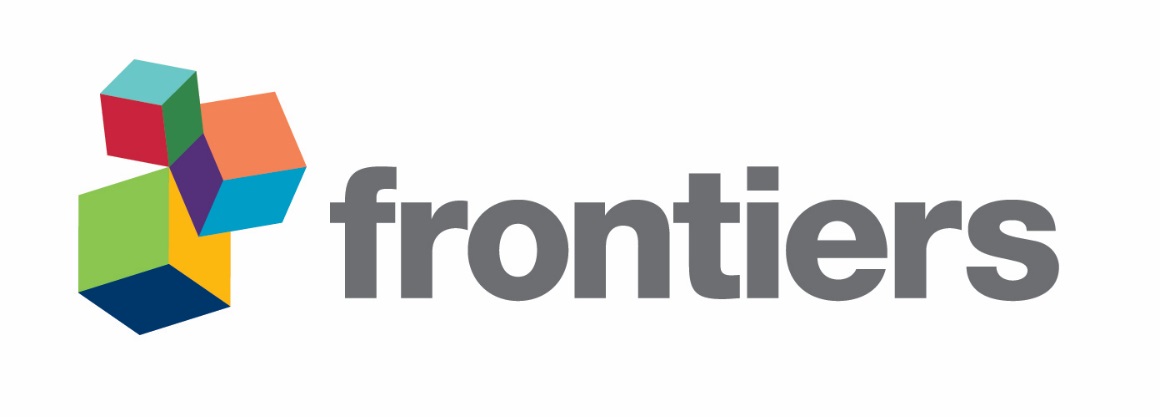
**
